# Seasonal Patterns of Mpox Index Cases, Africa, 1970–2021

**DOI:** 10.3201/eid3005.230293

**Published:** 2024-05

**Authors:** Camille Besombes, Festus Mbrenga, Ella Gonofio, Christian Malaka, Cedric-Stephane Bationo, Jean Gaudart, Manon Curaudeau, Alexandre Hassanin, Antoine Gessain, Romain Duda, Tamara Giles Vernick, Arnaud Fontanet, Emmanuel Nakouné, Jordi Landier

**Affiliations:** Institut Pasteur, Paris, France (C. Besombes, A. Gessain, R. Duda, T. Giles Vernick, A. Fontanet);; Institut Pasteur de Bangui, Bangui, Central African Republic (F. Mbrenga, E. Gonofio, C. Malaka, E. Nakouné);; Aix Marseille Univ, Marseille, France (C.-S. Bationo, J. Gaudart),; Institut de Recherche pour le Développement, Marseille (J. Landier);; National Museum of Natural History, Paris (M. Curaudeau, A. Hassanin);; Conservatoire National des Arts et métiers, Paris (A. Fontanet)

**Keywords:** Seasonal, mpox, viruses, zoonoses, Africa, monkey pox, zoonotic infections

## Abstract

Across 133 confirmed mpox zoonotic index cases reported during 1970–2021 in Africa, cases occurred year-round near the equator, where climate is consistent. However, in tropical regions of the northern hemisphere under a dry/wet season cycle, cases occurred seasonally. Our findings further support the seasonality of mpox zoonotic transmission risk.

Mpox, caused by monkeypox virus (MPXV), remains a neglected tropical zoonotic disease of forested Central and West Africa (*1*). Mpox epidemiology is poorly understood, and the MPXV animal reservoir remains unknown (*1*). Risk factors for zoonotic infection reportedly include direct or indirect contact with wildlife among subsistence activities in forests (*2*–*4*). Those characteristics may have evolved in West Africa because Nigeria reported sustained interhuman transmission of MPXV clade II in 2017, which led to the emergence of clade IIb and the global outbreak declared in May 2022 (*5*). In Central Africa at the time of our study, however, mpox cases remain typically linked to short chains of interhuman transmission after zoonotic spillover (*1*).

After 1990, reported case numbers increased sharply for Congo Basin/clade I, then a sharp increase in West African/clade II began in 2000 (*1*). The lack of systematic surveillance hinders investigative understanding of long-term temporal trends (*1*) and potential seasonality. Available index case time series suggested seasonal changes in risk and high-risk periods: outbreaks occurred predominantly in September in the Central African Republic (CAR) (*6*), and during June–August in different regions of Democratic Republic of the Congo (DRC) (*7*–*11*). We analyzed potential zoonotic transmission seasonality from reported mpox index cases in Africa during 1970–2021.

## The Study

We systematically analyzed peer-reviewed and gray literature reporting mpox (formerly monkeypox) index cases from zoonotic origin in Africa during 1970–2021. We extracted index case geographic localization and occurrence dates for temporal and spatial analysis. We used the PubMed query (“1970”[Date-Publication]: “2021/12/31”[Date-Publication]) AND monkeypox AND Africa (Appendix Figure 1).

We only included index cases defined as the first reported case presumed to result from zoonotic transmission in an epidemiologic outbreak investigation. We excluded cases outside Africa and those related to secondary interhuman transmission. We also excluded cases without PCR, viral isolation or culture, or electron microscopy confirmation; and cases without onset month or geographic localization (Appendix Figure 2).

We defined index sites as locations with >1 index case. We extracted remotely sensed meteorologic (precipitation, daytime and nighttime temperature), topographic (altitude and slope), land use–land cover data, and fire occurrence data using a 10-km radius buffer zone around each site (Appendix Table 1).

We conducted unsupervised clustering to regroup sites into climate and seasonality (hereafter climate), landscape, and environment profiles. For climate profiles, we included the average cumulative rainfall, daytime and nighttime temperature, and fire index values (Appendix Table 1) for each month in a principal component analysis. We performed hierarchical clustering on principal components, including 99% of dataset inertia, by using R version 4.3 (The R Foundation for Statistical Computing, https://www.r-project.org) and FactoMineR package (https://cran.r-project.org/web/packages/FactoMineR/index.html). We grouped sites by maximizing within-group similarity and between-groups difference. Following the same approach, we used variables describing the percentage of each buffer occupied by each land use–land cover class (e.g., evergreen closed forest, cropland) and topographic variables to obtain landscape profiles. Finally, we combined the 2 sets of variables to generate combined environment profiles (Appendix).

We used the Kruskall-Wallis test to first compare the distribution of latitudes by month of index case occurrence and then to compare months of occurrence according to site environmental characteristics defined by each profile. Using months as a quantitative variable enabled comparison of periods of the year rather than specific months. Sensitivity analyses determined whether the association remained when restricting the analysis to clade II and to recent (2001–2021) cases (Appendix).

We identified 208 index cases: 145 reported from 53 peer-reviewed articles, 26 from 35 gray literature sources, and 37 from CAR national surveillance data (Appendix Figure 2). After exclusion criteria, we retained 133 index cases from 113 sites; 64% were reported from 2000 onward (Appendix Figure 2). Clade I represented 86% of index cases, and clade II represented 13%. DRC accounted for 44% of index cases, and CAR accounted for 33% (Table).

**Table:** Location and case characteristics in a study of seasonal patterns of mpox index cases, Africa, 1970–2021

Variable	No. (%) cases, n = 133
Country	
Cameroon	7 (5.3)
Central African Republic	44 (33)
Democratic Republic of the Congo	58 (44)
Gabon	2 (1.5)
Ivory Coast	1 (0.8)
Liberia	5 (3.8)
Nigeria	3 (2.3)
Republic of Congo	8 (6.0)
Sierra Leone	4 (3.0)
South Sudan	1 (0.8)
Timeframe	
1970–1980	35 (26)
1981–1990	8 (6.0)
1991–2000	5 (3.8)
2001–2010	16 (12)
2011–2021	69 (52)
Strain	
Clade I	115 (86)
Clade II	17 (13)
Unknown	1 (0.8)
Outbreak month	
January	15 (11)
February	20 (15)
March	12 (9.0)
April	6 (4.5)
May	7 (5.3)
June	5 (3.8)
July	3 (2.3)
August	14 (11)
September	16 (12)
October	9 (6.8)
November	14 (11)
December	12 (9.0)
Climate or seasonality profile	
Northern hot, wet-dry	23 (17)
Northern cool, wet-dry	47 (35)
Equatorial cool	44 (33)
Southern hot, wet-dry	14 (11)
Other	5 (3.8)
Landscape profile	
Open forest + river or wetlands	23 (17)
Evergreen closed forest	68 (51)
Deciduous forest, closed or open	26 (20)
Grassland + hills	11 (8.3)
Other	5 (3.8)
Combined environmental profile	
Evergreen closed forest + warm night	80 (60)
Evergreen open forest + high precipitation	10 (7.5)
Southern tropical, grassland + hills	14 (11)
Deciduous forest + hot month of January	24 (18)
Other	5 (3.8)

**Figure 1:**
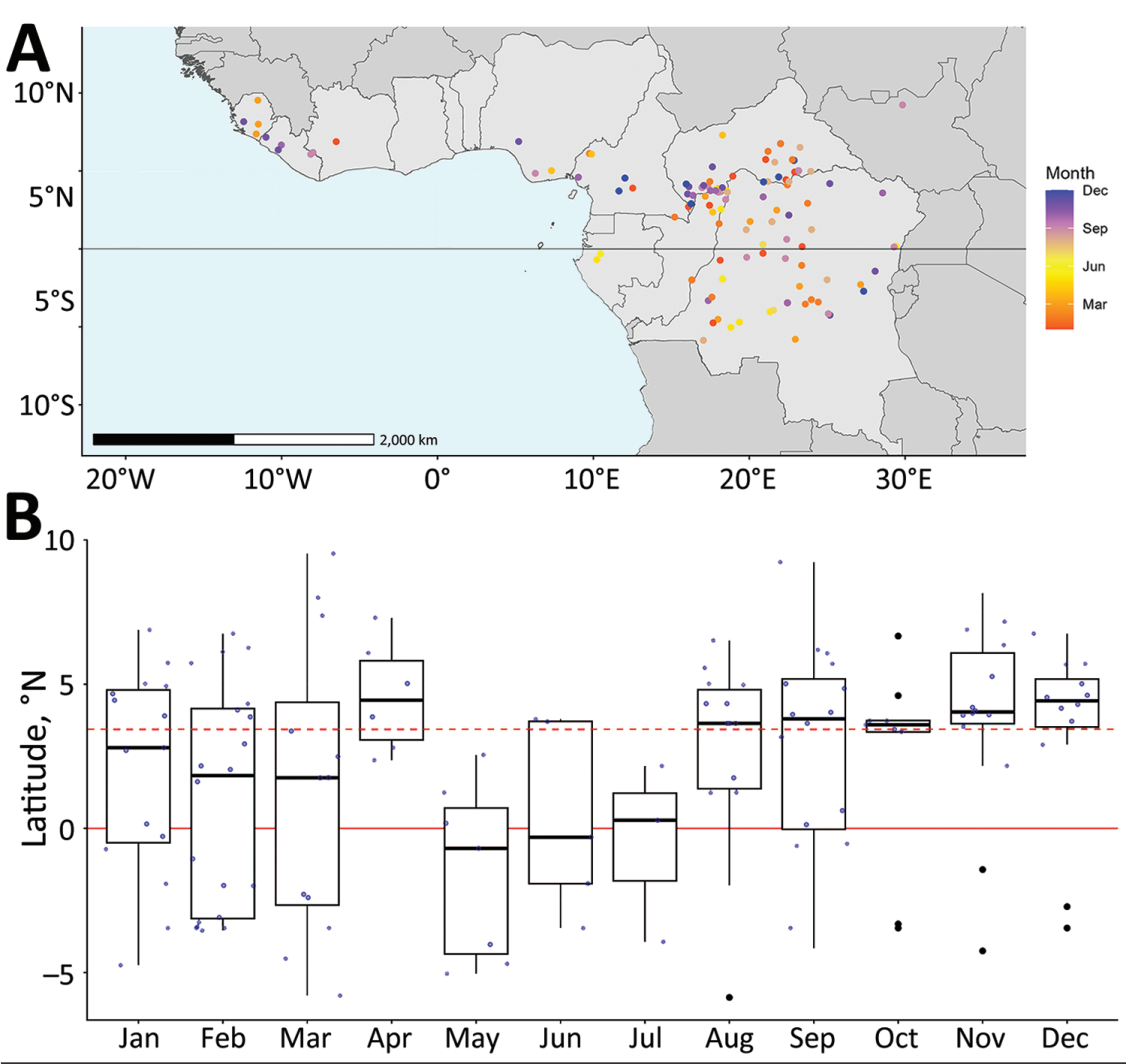
Distribution of cases in study of seasonal patterns of Mpox index cases, Africa, 1970–2021. A) Map of mpox index case sites and month recorded; black horizontal line indicates equator. B) Boxplot of latitude of index case according to month of occurrence. Red dashed line indicates the median latitude of index cases; red solid line indicates the equator (latitude = 0). The thick black line indicates the median latitude, and the box tops and bottom indicate the upper quartile (above) and the lower quartile (below). Whiskers extend from upper quartile to upper quartile+1.5 interquartile range and extend down from lower quartile to lower quartile−1.5 interquartile range. Solid black dots signify outlying observations beyond whiskers range; blue circles identify all other observations.

Index cases occurred at a median latitude of 3.44°N (range: −5.87 to 9.53). Index cases latitudes differed significantly across months (p = 0.0354). During January–July (April excluded), index cases mostly occurred <3.44°N, whereas during August–December, most cases occurred >3.44°N (Figure 1).

**Figure 2:**
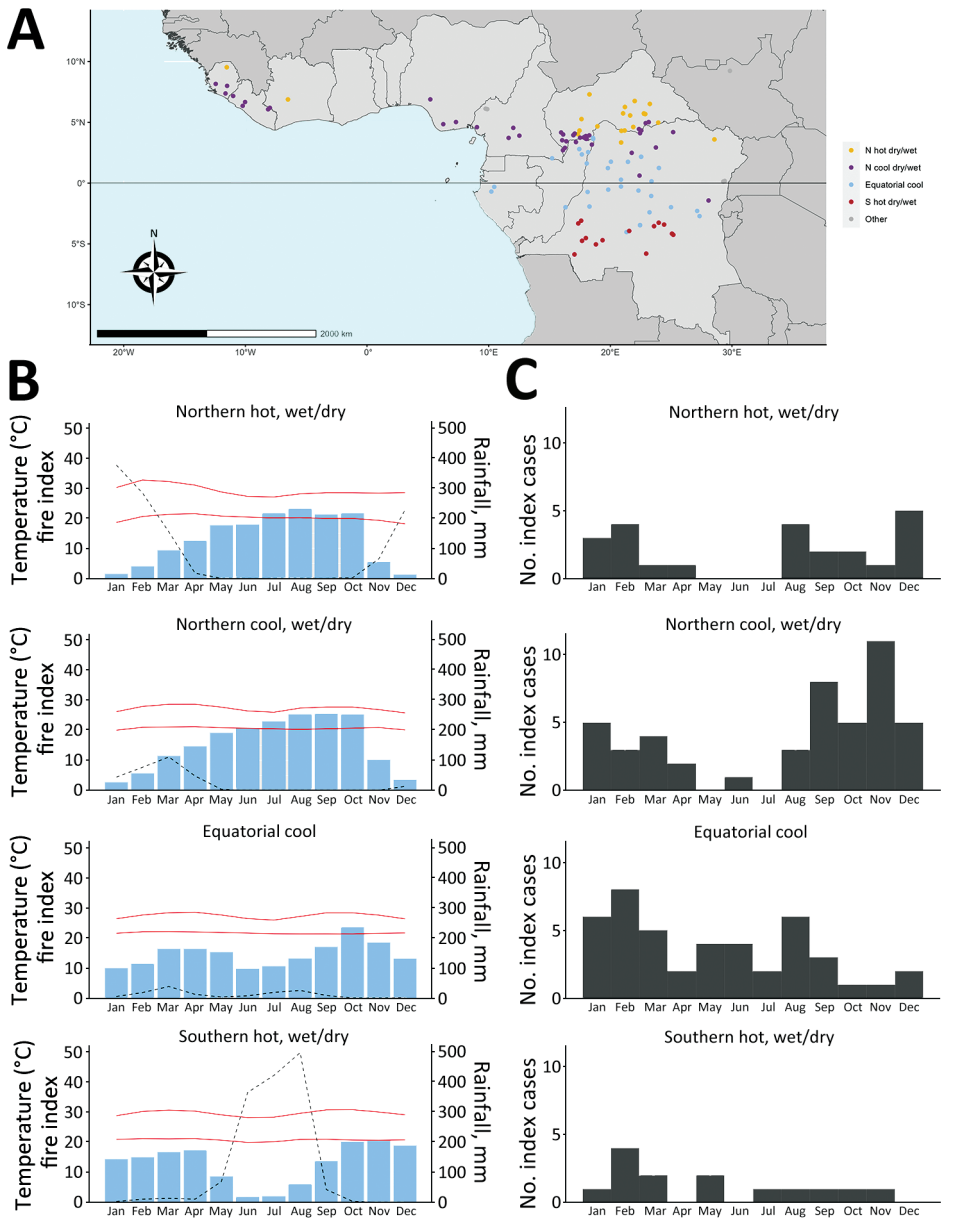
Seasonal distribution of mpox index cases according to the climate profile in Africa, 1970–2021. A) Climate/seasonal profile by site. B) Average monthly rainfall, temperature, and fire index (dotted line) for each climate/seasonal profile. C) Distribution of outbreak index cases by month for each climate/seasonal profile.

We excluded 4 index sites at high altitude (>1,000 m) and 1 Sahelian index site because rare mpox occurrence prevented seasonality characterization in those settings. The other 108 sites clustered into 4 climate profiles. The equatorial cool profile had temperatures <30°C and day-night amplitude <10°C, rainfall across all months, and site latitudes ranging from −4.00° to 4.00°N (Figure 2, panels A, B). The northern cool wet-dry profile had similarly low temperatures and amplitude, a dry season during December–February, and site latitudes in the northern hemisphere. The northern hot wet-dry profile displayed temperatures >30°C during the hottest months, a marked dry season during November–March, and latitude sites in the northern hemisphere. The southern hot wet-dry profile had similarly hot temperatures, a dry season in May–August, and latitude sites in the southern hemisphere (Figure 2, panels A, B). Index cases occurred mostly in the equatorial cool (33%), northern cool wet-dry (35%), and northern hot wet-dry profiles (17%) (Table).

Cases occurred throughout the year in the equatorial cool profile and varied seasonally in the 2 northern wet-dry profiles; cases were nearly absent during April–July (Figure 2, panel C). Seasonality analysis remained inconclusive in the southern hot wet-dry profile, which had low sample size (n = 14). The distribution of index case months was significantly different between climate profiles (p = 0.004). That association persisted in sensitivity analyses (Appendix Tables 2, 3). Landscape and combined environment profiles had poorer association with the month of an index case (Appendix Tables 2, 3. Figures 4, 6, 7).

## Conclusions

We showed that the monthly distribution of mpox index cases varied with latitude and was associated with specific climates across the main ecologic mpox niche, excluding high altitude and Sahelian sites. We identified high-risk and low-risk periods across the year in sites located in northern hemisphere climates with alternating dry and wet seasons (>50% of index cases analyzed).

A potential high-risk season occurred during August–March, spanning the last 3 months of the rainy season and all the dry season. That finding suggests complex drivers likely related to human and wildlife ecology. Various seasonal activities can increase human contact with wildlife. During the wet season, human populations settle in forest camps to collect edible caterpillars (*6*), a major source of protein and income (*12*). Hunting and trapping activities, generally conducted year round, intensify during the dry season (*10*). Likewise, dry season slash-and-burn activities tend to drive mammals, notably rodents, toward food resources in neighboring fields, resulting in closer contact with humans.

Multiple obstacles, including access to healthcare, hinder exhaustive mpox reporting from endemic regions. Therefore, this analysis relied on a limited series of well-characterized index cases. Such data and indirect approaches were used extensively to study mpox ecologic niches (*13*) and emerging diseases with similar surveillance gaps (e.g., Ebola virus) (*14*). Our conclusion that MPXV zoonotic transmission risk could be seasonal in regions under a dry-wet season cycle warrants further investigation.

Ongoing climate and environmental changes could exacerbate potential underlying seasonal drivers of human MPXV exposure. Determining whether specific seasons or periods bring greater risk for human transmission can improve prevention and surveillance initiatives and contribute to identifying animal reservoirs. For this, a genuine One Health approach is crucial (*15*).

Appendix More information is available for seasonal patterns of mpox index cases, Africa, 1970–2021.

## References

[1] Bunge EM, Hoet B, Chen L, Lienert F, Weidenthaler H, Baer LR, et al. The changing epidemiology of human monkeypox-A potential threat? A systematic review. PLoS Negl Trop Dis. 2022;16:e0010141. 10.1371/journal.pntd.0010141 35148313 PMC8870502

[2] Hutin YJ, Williams RJ, Malfait P, Pebody R, Loparev VN, Ropp SL, et al. Outbreak of human monkeypox, Democratic Republic of Congo, 1996 to 1997. Emerg Infect Dis. 2001;7:434–8. 10.3201/eid0703.017311 11384521 PMC2631782

[3] Patrono LV, Pléh K, Samuni L, Ulrich M, Röthemeier C, Sachse A, et al. Monkeypox virus emergence in wild chimpanzees reveals distinct clinical outcomes and viral diversity. Nat Microbiol. 2020;5:955–65. 10.1038/s41564-020-0706-0 32341480

[4] Narat V, Alcayna-Stevens L, Rupp S, Giles-Vernick T. Rethinking human-nonhuman primate contact and pathogenic disease spillover. EcoHealth. 2017;14:840–50. 10.1007/s10393-017-1283-4 29150826

[5] Forni D, Cagliani R, Molteni C, Clerici M, Sironi M. Monkeypox virus: The changing facets of a zoonotic pathogen. Infect Genet Evol. 2022;105:105372. 10.1016/j.meegid.2022.105372 36202208 PMC9534092

[6] Besombes C, Mbrenga F, Schaeffer L, Malaka C, Gonofio E, Landier J, et al. National monkeypox surveillance, Central African Republic, 2001–2021. Emerg Infect Dis. 2022;28:2435–45. 10.3201/eid2812.220897 36328951 PMC9707566

[7] Heymann DL, Szczeniowski M, Esteves K. Re-emergence of monkeypox in Africa: a review of the past six years. Br Med Bull. 1998;54:693–702. 10.1093/oxfordjournals.bmb.a011720 10326294

[8] Breman JG, Kalisa-Ruti, Steniowski MV, Zanotto E, Gromyko AI, Arita I. Human monkeypox, 1970-79. Bull World Health Organ. 1980;58:165–82. 6249508 PMC2395797

[9] Jezek Z, Grab B, Szczeniowski MV, Paluku KM, Mutombo M. Human monkeypox: secondary attack rates. Bull World Health Organ. 1988;66:465–70. 2844429 PMC2491159

[10] Mandja BM, Brembilla A, Handschumacher P, Bompangue D, Gonzalez JP, Muyembe JJ, et al. Temporal and spatial dynamics of monkeypox in Democratic Republic of Congo, 2000–2015. EcoHealth. 2019;16:476–87. 10.1007/s10393-019-01435-1 31410720

[11] Ježek Z, Fenner F. Human monkeypox. Basel, Switzerland: S. Karger AG; 1988..

[12] Malaisse F, Lognay G. Edible caterpillars from tropical Africa. In: Motte-Florac E, Thomas JMC, eds. “Insects” an oral tradition [in French]. Paris-Louvain: Peeters-SELAF; 2003. pp 279–304.

[13] Levine RS, Peterson AT, Yorita KL, Carroll D, Damon IK, Reynolds MG. Ecological niche and geographic distribution of human monkeypox in Africa. PLoS One. 2007;2:e176. 10.1371/journal.pone.0000176 17268575 PMC1769466

[14] Lee-Cruz L, Lenormand M, Cappelle J, Caron A, De Nys H, Peeters M, et al. Mapping of Ebola virus spillover: Suitability and seasonal variability at the landscape scale. PLoS Negl Trop Dis. 2021;15:e0009683. 10.1371/journal.pntd.0009683 34424896 PMC8425568

[15] Mandja BA, Handschumacher P, Bompangue D, Gonzalez JP, Muyembe JJ, Sauleau EA, et al. Environmental drivers of monkeypox transmission in the Democratic Republic of the Congo. EcoHealth. 2022;19:354–64. 10.1007/s10393-022-01610-x 36029356

