# Supplementary material for: Seasonal Patterns of Mpox Index Cases, Africa, 1970–2021
Source: Emerg Infect Dis. 2024 May;30(5):1017–21. doi: 10.3201/eid3005.230293 (PMC11060470; doi:10.3201/eid3005.230293)
Supplement: Appendix — More information is available for seasonal patterns of mpox index cases, Africa, 1970–2021. [file 23-0293-Techapp-s1.pdf]

Article DOI: http://doi.org/10.3201/eid3005.230293

EID cannot ensure accessibility for supplementary materials supplied by authors. Readers who have difficulty accessing supplementary content should contact the authors for assistance.

Seasonal Patterns of Mpox Index Cases, Africa, 1970–2021

Appendix

Mpox Seasonality Analysis

Definition: An index case corresponds to the first reported human case presumed to result from zoonotic transmission after an epidemiologic outbreak investigation.

Systematic Review and Index Case Selection Flowcharts

Sources of gray literature were the Web sites of the World Health Organization (WHO disease outbreak news), the Weekly Bulletin on Outbreaks and other Emergencies, United States Centers for Disease Control and Prevention (CDC), Morbidity and Mortality Weekly Record (MMWR), Africa CDC, Nigeria CDC, ProMed mail and other sources such as Relief web, Outbreak news today or report of outbreak on regional radio confronted to ProMed-mail information (Appendix Figures 1, 2).

Environmental Data Sources

We extracted all environmental data from various open access sources aggregated by the Google Earth Engine platform using the interface provided by R package {rgee} (*1*–*5*). Specifically for Copernicus Global Land Cover dataset, landcover classes retained were: Shrub vegetation (category coded 20), Herbaceous vegetation (code 30), Cropland (code 40), Urban/built-up (code 50), Bare soil (code 60), Waterbodies or wetlands (combining code 80 and 90), Closed forest, evergreen broad leaf (112), Closed forest, deciduous broad leaf (114), Other

closed forest (116), Open forest, evergreen broad leaf (122), Open forest, deciduous broad leaf (124), Other open forest (126) (*5*) (Appendix Table 1).

Environment Profile Generation Methods

We performed multistep nonsupervised analysis (clustering) to classify index case sites according to their environmental features (climate/seasonality, landscape, or combined). The overall method consisted in hierarchical clustering on the principal components of a principal component analysis (PCA) (*6,7*). The first step was a PCA, which is an exploratory method that considers the relationship between variables and reduces complex datasets into fewer dimensions. We performed PCA by using continuous variables. Active variables included:

- For climate/seasonality profile: average monthly cumulative rainfall, daytime temperature, nighttime temperature, fire index from January to December. This profile combine mainly climate-related parameters (temperatures and rainfall), but also fire index which is related to both climate (vegetation has to be dry to burn) but also to human activities (fires are often started by humans, for example to clear and fertilize fields at the start of the planting season).
- For landscape: percentage of surface occupied by Shrub vegetation (category coded 20), Herbaceous vegetation (code 30), Cropland (code 40), Urban/built-up (code 50), Bare soil (code 60), Waterbodies or wetlands (combining code 80 and 90), Closed forest, evergreen broad leaf (112), Closed forest, deciduous broad leaf (114), Other closed forest (116), Open forest, evergreen broad leaf (122), Open forest, deciduous broad leaf (124), Other open forest (126); min, max, mean, median, standard deviation for altitude and slope.
- For combined environment: all variables in the two previous points

To reduce basal noise and ensure a more stable classification, we retained the principal components that summarized 99% of the data. We performed hierarchical ascendant classification on the first 30, 15, and 37 principal components' coordinates, for respectively climate/seasonality, landscape, and combined environment. Each approach provided classes independent of the month of mpox index case occurrence.

Supplementary Results

In addition to climate profiles described in the main article (Appendix Figure 4, panel A), we identified 4 landscape profiles (after excluding the same 5 sites, Appendix Figure 4, panel B). Open forest combined with river or wetland presence (Open+River/wet) occurred mainly in sites bordering the Gulf of Guinea, but also in the Congo Basin. Evergreen closed forest landscape (Evergr. Closed) was dominant in the Congo Basin Forest sites, near the Equator. Deciduous forest landscapes (open or closed) corresponded to inland outbreak sites in the tropical area, at latitudes higher than 5°N or S. Grassland+hill landscape corresponded to sites located around 5°S (Appendix Figures 3, 4).

Regarding the combined environment clustering (landscape+climate, Appendix Figure 4, panel C), profiles combined roughly the climate and landscape profiles and led to one large equatorial profile, regrouping evergreen closed forest with narrow temperature variations and characterized by warmer night temperatures. The clade II/West African outbreak sites were majoritarily in a profile corresponding to evergreen open forest with abundant cumulative precipitation (Ev.open+rain). Sites located around 5°N inland grouped in a deciduous forest group, with hotter temperatures in January (Decid.+hot Ja), while those located around 5°S inland grouped in a grassland+hill profile, both largely similar to the landscapes and climates (Appendix Figure 4, panel A).

Distribution of Mpox Outbreak Months

For all further analyses, we removed the 4 mpox index cases corresponding to the 4 mountains sites (2 in Cameroon and 2 in DRC) located at altitudes >1000m leading to specific climate and topography and 1 index case which occurred in South Sudan, characterized by outlying climate profile (Sahelian) (Appendix Figures 5–7). Combined environmental profile obtained by including climate and landscape data in a single PCA+HCPC.

Sensitivity Analyses on the Association Between Climate/Seasonality Profile and Month of Index Case Occurrence

To test the robustness of the association between climate and month of occurrence of mpox index cases, we conducted several analyses. First, we analyzed all outbreaks across 3 main periods of mpox incidence: 1970–1980 during the smallpox eradication effort; 1981–2000 when outbreaks were only reported in small numbers; and 2001–2021, corresponding to the current context. We then focused specifically on 2001–2021. The main analysis grouped clade I and clade II index cases. There could be differences between the transmission contexts of the two clades, and clade II added a wide geographic area but a limited number of events.

Because our analysis aimed to present hypotheses of current major zoonotic transmission, we studied whether the association identified remained valid for the recent period only (2001–2021) and when considering only clade I cases (which restricted the analysis to central African countries). Since there were only 2 index cases recorded in the Southern hot wet/dry profile over the 2001–2021 period, we also conducted a sensitivity analysis excluding this profile ("After 2000+Clade I, Southern hot wet/dry profile excluded").

We applied these conditions individually or in combination for the 3 profiles defined (climate/seasonality, landscape, and combined environment). The association between climate/seasonality and month of occurrence remained throughout an increasingly homogenous index case sample (Appendix Table 2). For landscapes profile, the association was not significant over the entire set of outbreaks but became significant when excluding events before 2001. This likely led to convergence between landscape and climate/seasonality profiles.

In a second step, we studied whether the bimodal distribution observed in Northern Tropical profiles (cases identified from January to March and from August to December) could have influenced the result of the statistical test. Indeed, in Appendix Figure 8, we presented median and IQR for bimodal distributions in Northern Tropical profiles.

To limit this issue, we defined the start of the mpox epidemiologic year in August, i.e., after minimum occurrence in July (Appendix Figure 9). We replicated the analysis of the distribution of index case month by "epidemiologic year" starting in August, i.e., ending with minimum occurrence in July (Appendix Table 3). This enabled us to account for the fact that

cases in December and January belonged to the same period of the year (i.e., dry season in Northern tropical profiles). We thus confirmed that the difference in distribution identified in Appendix Table 2 was not an artifact related to our analysis using calendar months (Appendix Table 3).

References

1. Funk C, Peterson P, Landsfeld M, Pedreros D, Verdin J, Shukla S, et al. The climate hazards infrared precipitation with stations—a new environmental record for monitoring extremes. Sci Data. 2015;2:150066 PubMed.https://doi.org/10.1038/sdata.2015.66

2. Wan Z, Hook S, Hulley G. 2021. MODIS/Terra Land Surface Temperature/Emissivity Daily L3 Global 1km SIN Grid V061 [Data set]. NASA EOSDros Inf Serv. Land Processes Distributed Active Archive Center. https://doi.org/10.5067/MODIS/MOD11A1.061

3. MODIS/Aqua+Terra Thermal Anomalies/Fire locations 1km V006 and V0061 Standard (Vector data) distributed by LANCE FIRMS https://doi.org/10.5067/FIRMS/MODIS/MCD14ML

4. Farr TG, Rosen PA, Caro E, Crippen R, Duren R, Hensley S, et al. The shuttle radar topography mission. Rev Geophys. 2007;45:RG2004.https://doi.org/10.1029/2005RG000183

5. Buchhorn M, Lesiv M, Tsendbazar N-E, Herold M, Bertels L, Smets, B. Copernicus Global Land Cover Layers-Collection 2. 2020;12(6):1044. https://doi.org/10.3390/rs12061044

6. Lê S, Josse J, Husson F. FactoMineR: an R package for multivariate analysis. J Stat Softw. 2008;25:1–18.https://doi.org/10.18637/jss.v025.i01

7. Griffiths K, Moise K, Piarroux M, Gaudart J, Beaulieu S, Bulit G, et al. Delineating and Analyzing Locality-Level Determinants of Cholera, Haiti. Emerg Infect Dis. 2021;27:170–81. PubMedhttps://doi.org/10.3201/eid2701.191787

Appendix Table 1. Environmental data description

Profile	Data description	Data source	Indicator	N variables	Reference no.
Climate/seasonality	Accumulated precipitation (mm)	CHIRPS	Monthly average, 1981–2021	12	(*1*)
	Daytime average Temperature (°C)	MODIS LST	Monthly average, 2000–2021	12	(*2*)
	Nighttime average Temperature (°C)	MODIS LST	Monthly average, 2000–2021	12	(*2*)
	Fire index (surface burned)	FIRMS	Monthly sum of burned surface 2000–2021	12	(*3*)
Landscape	Altitude (m)	SRTM 30m	Mean, median, min, max, standard deviation	5	(*4*)
	Slope (%)	SRTM 30m	Mean, median, min, max, standard deviation	5	(*4*)
	Land-use-Landcover	Copernicus Global Land Cover	% of Buffer surface in each land-use-landcover class	12	(*5*)
Combined environment	Include all variables from the Climate/Seasonality and Landscape profiles			70	

Appendix Table 2. Results of Kruskall-Wallis test comparing the distribution of index case month of occurrence according to climate/season, landscape and combined environment profile, sorted by calendar year (starting in January). We assessed the robustness of the association in increasingly well characterized and homogenous subsets of data

Conditions	No. index cases included	Climate/ Seasonality	Landscape	Combined environment
All outbreaks	128	0.0042759	0.3364357	0.1751695
Clade I only	112	0.0089514	0.1172652	0.3194001
After 2000 only	81	0.0122368	0.0123876	0.3971197
After 2000+Clade I	73	0.0087610	0.0030186	0.3065604
After 2000+Clade I, Southern hot wet/dry profile excluded	71	0.0060040	0.0044488	0.2895289

Appendix Table 3. Results of Kruskall-wallis test comparing the distribution of mpox index case month of occurrence according to climate/season, landscape and combined environment profile, after ordering the months by mpox epidemiologic year. The red dashed lines indicate the low-risk period in Northern tropical-wet profile. We assessed the robustness of the association in increasingly well characterized and homogenous subsets of data

Conditions	No. index cases included	Climate/ Seasonality	Landscape	Combined environment
All outbreaks	128	0.0047424	0.2001057	0.3011334
Clade I only	112	0.0065628	0.0484065	0.1138376
After 2000 only	81	0.0900866	0.2737394	0.6585237
After 2000+Clade I	73	0.0699108	0.1016864	0.4085023
After 2000+Clade I, Southern hot wet/dry profile excluded	71	0.0453245	0.1288348	0.3711843

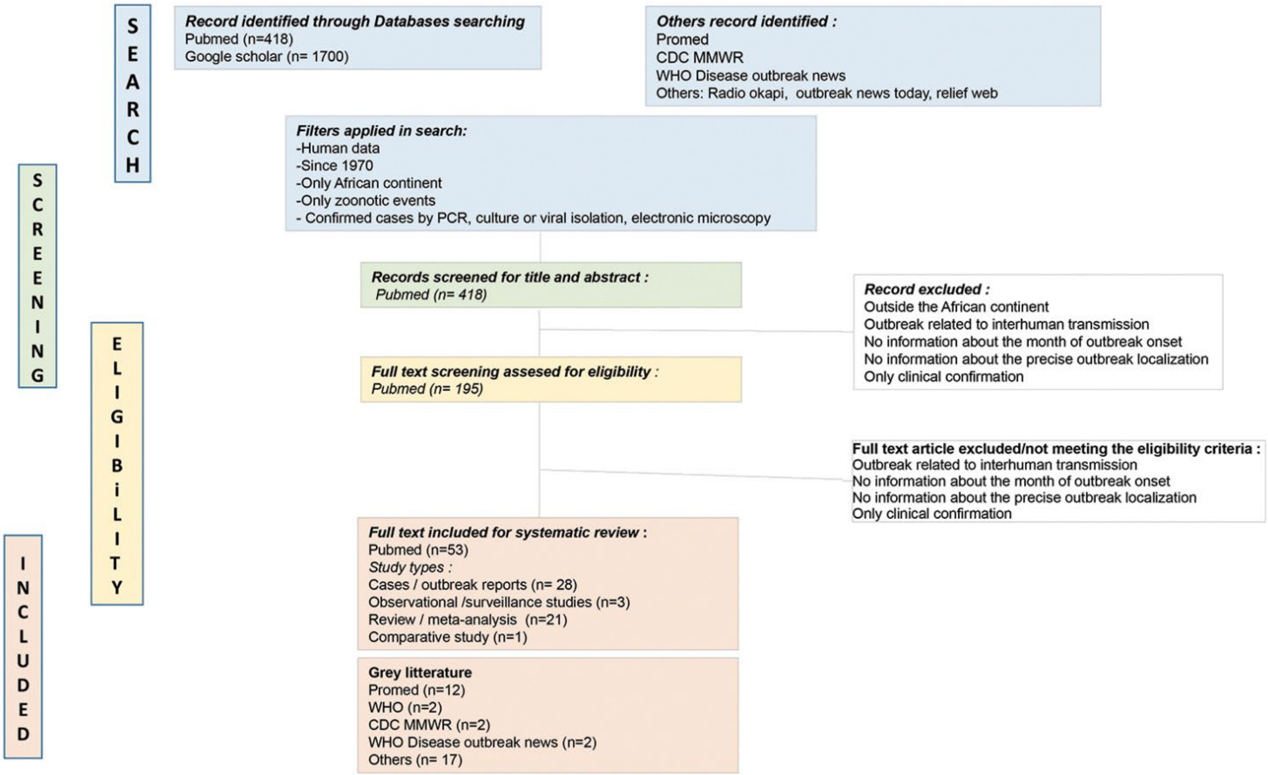

Appendix Figure 1. Systematic review flowchart.

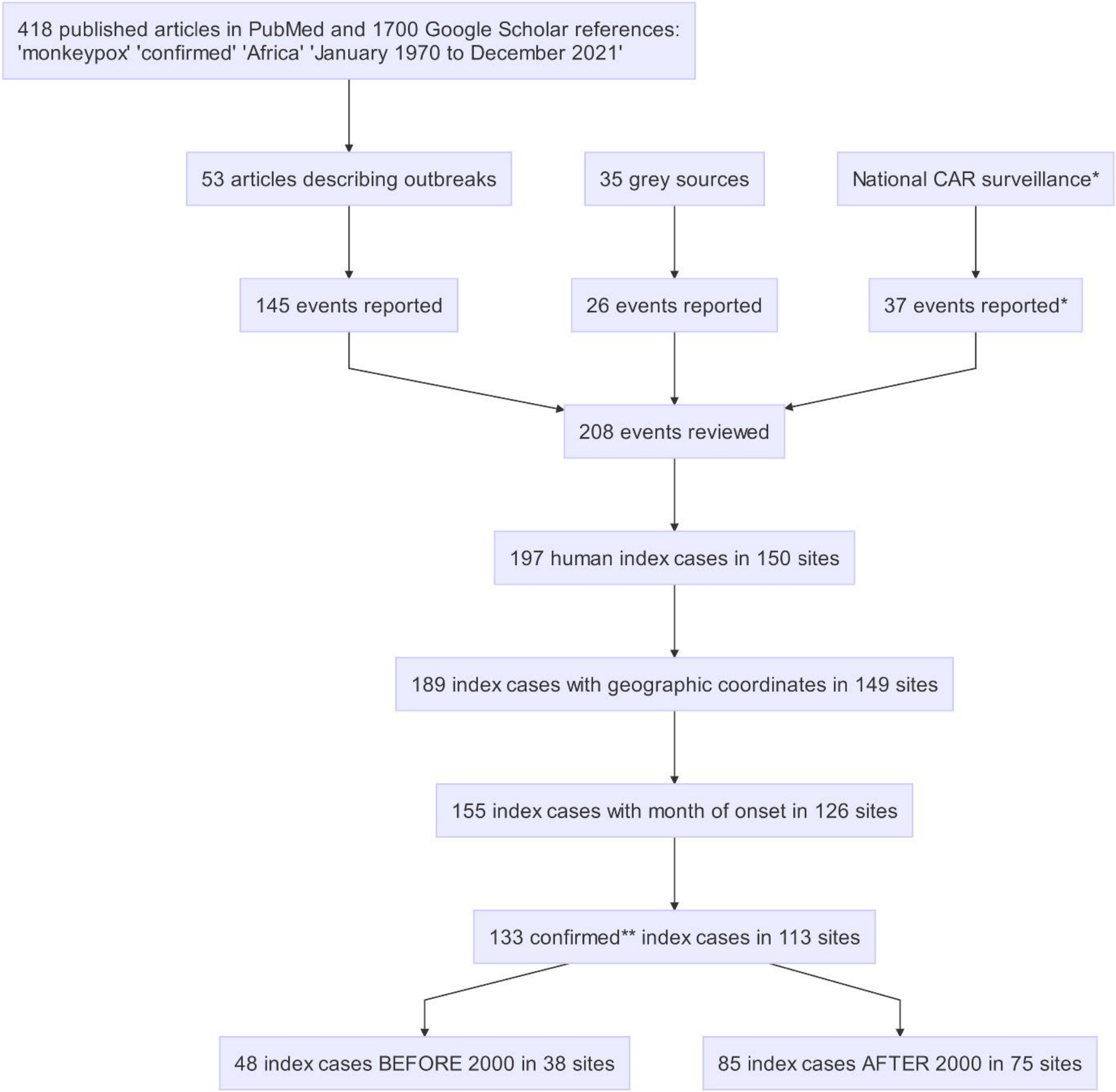

Appendix Figure 2. Detailed flowchart for mpox outbreaks included from the literature review, scientific and gray sources and National Central African Republic (CAR) mpox surveillance provided by the Institut Pasteur de Bangui and the Ministry of Public Health and Population of CAR. *Of 37 unpublished events at the time of review, 26 have been subsequently published by C. Besombes et al., Emerging Infectious Diseases, December 2022 (https://doi.org/10.3201/eid2812.220897). **Confirmation by robust methods only: PCR, viral isolation/culture, or electron microscopy according to inclusion criteria.

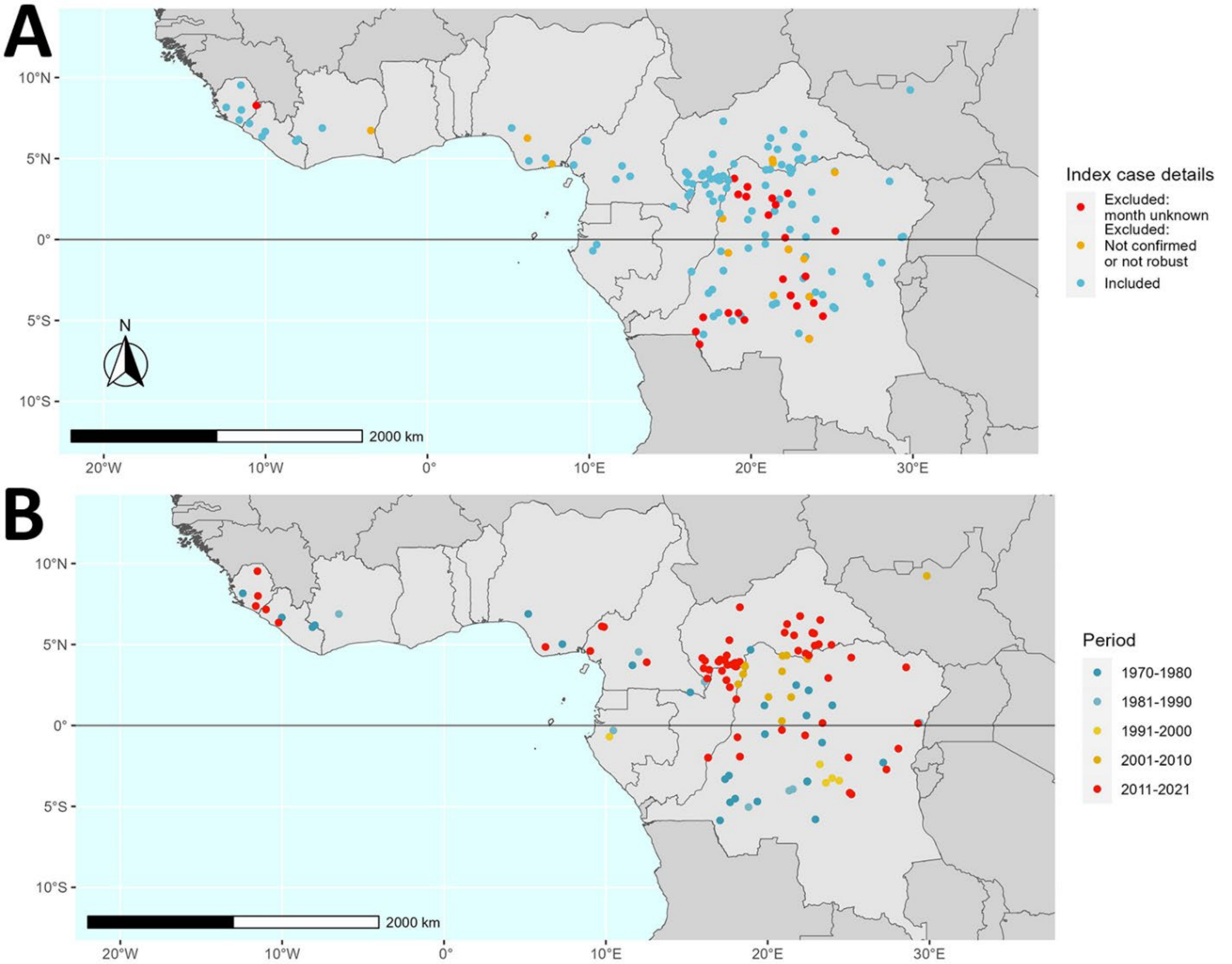

Appendix Figure 3. Map of mpox outbreak sites distribution. A) Human mpox index case sites (n = 149) with geographic coordinates, displaying sites included (blue) or excluded due to unreported month of occurrence (red) or not confirmed by PCR, viral isolation/culture, or electron microscopy (orange). B) Period of outbreak occurrence (n = 113 included sites).

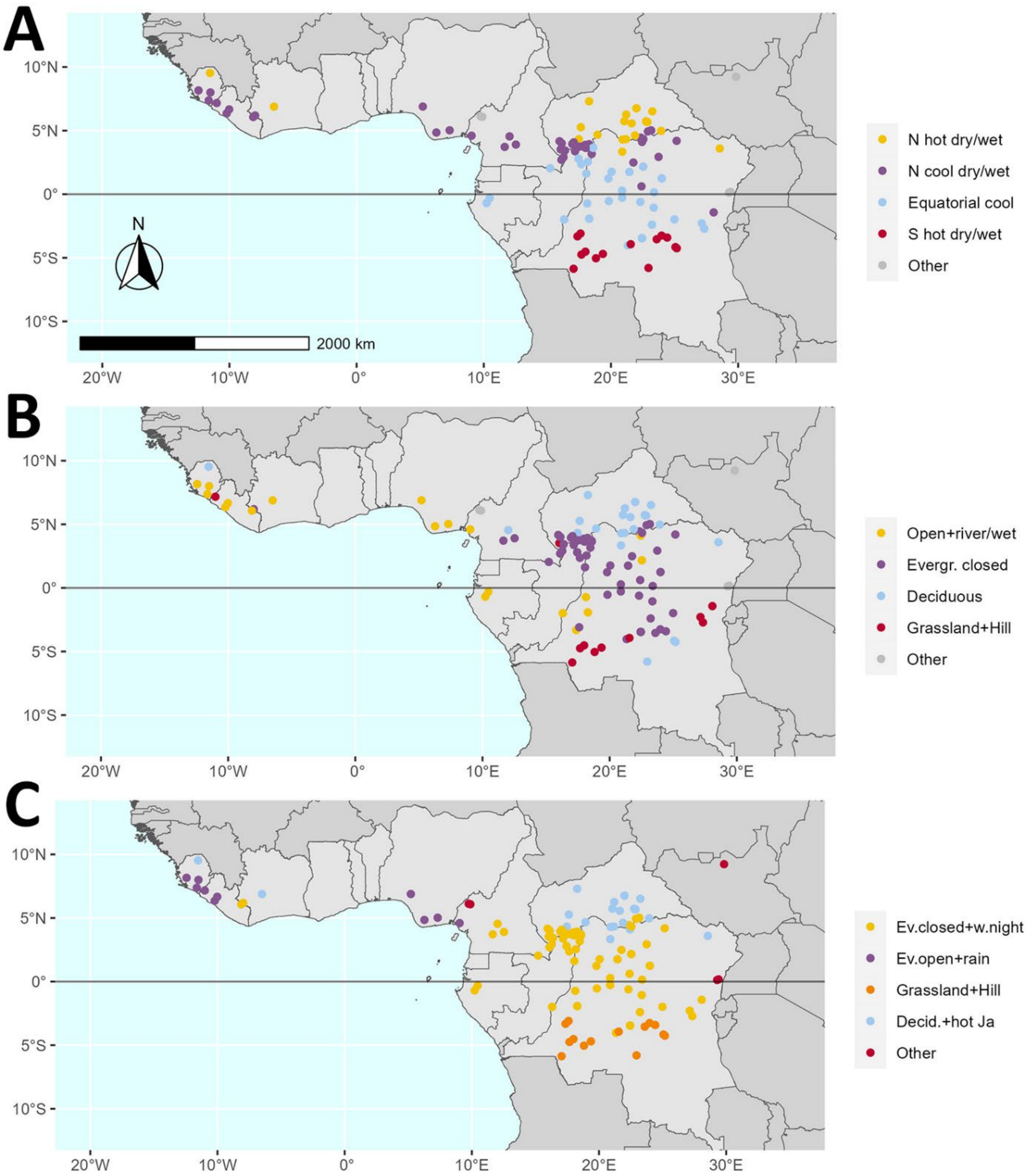

Appendix Figure 4. Maps of mpox outbreak site distributions. A) Climate/seasonality profile. B) Landscape profile. Open+river/wet, open forest + river/wetlands; Evergr. Closed, Evergreen closed forest; Deciduous, deciduous forest; Grassland+Hills; Other, 5 excluded sites. C) Combined seasonal climate and landscape profile. Ev.closed+w.night, evergreen closed forest + warm nights; Ev.open+rain, evergreen open forest + high precipitation volumes; Grassland+Hill, Southern Grassland+Hills; Decid.+hot Ja, deciduous forest + hot month of January; Other, 5 excluded sites.

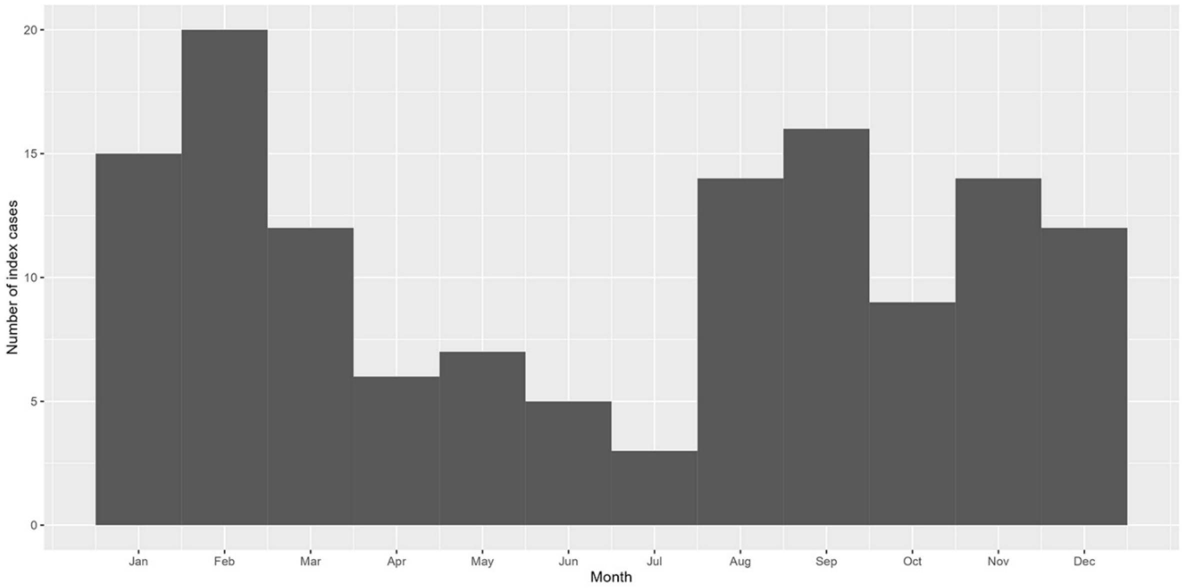

Appendix Figure 5. Distribution of mpox outbreaks by month of occurrence, n = 133 outbreaks.

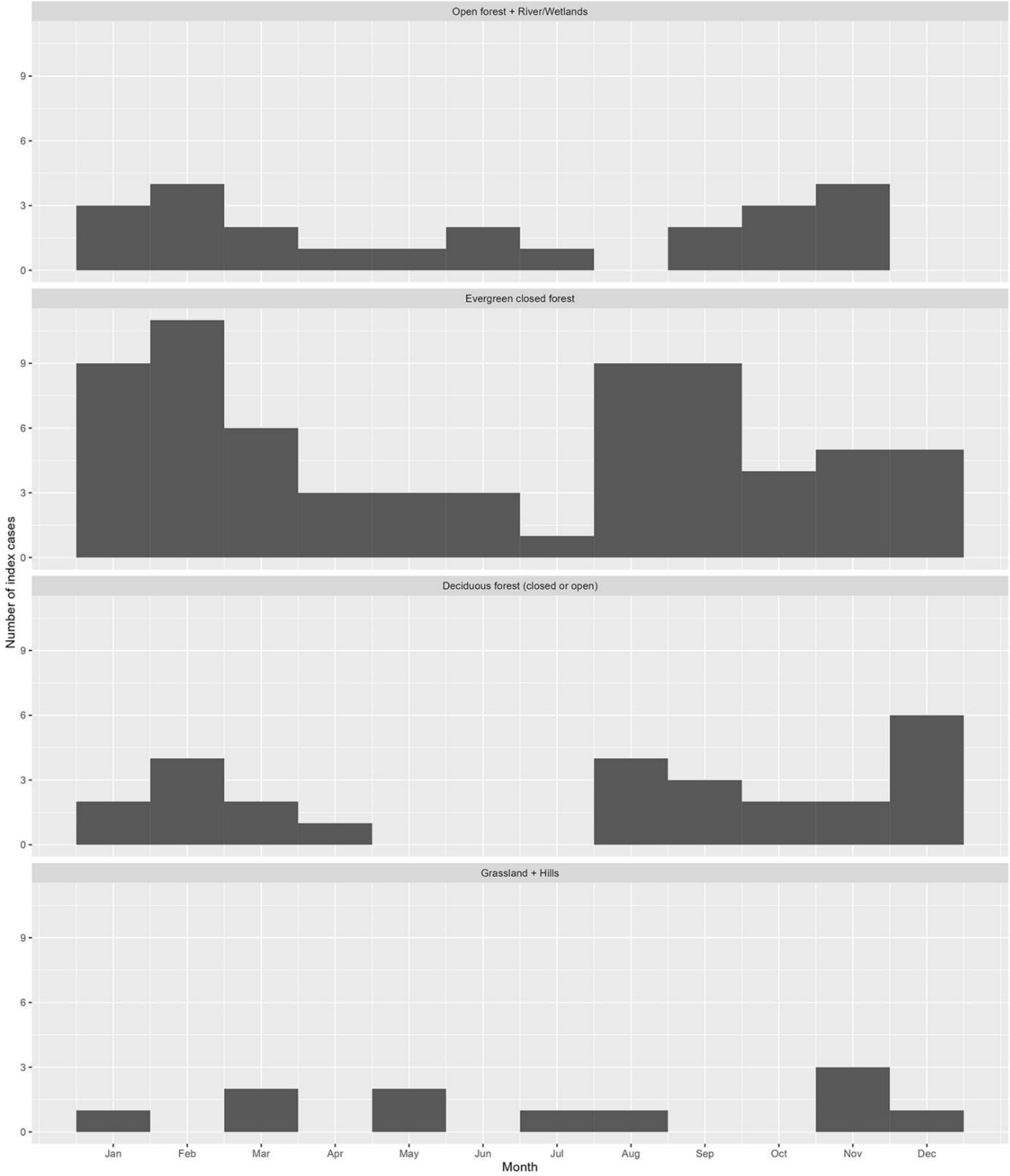

Appendix Figure 6. Distribution of mpox outbreaks by months, according to landscape profile, n = 128 index cases (after excluding 4 high altitude and 1 Sahelian climate locations).

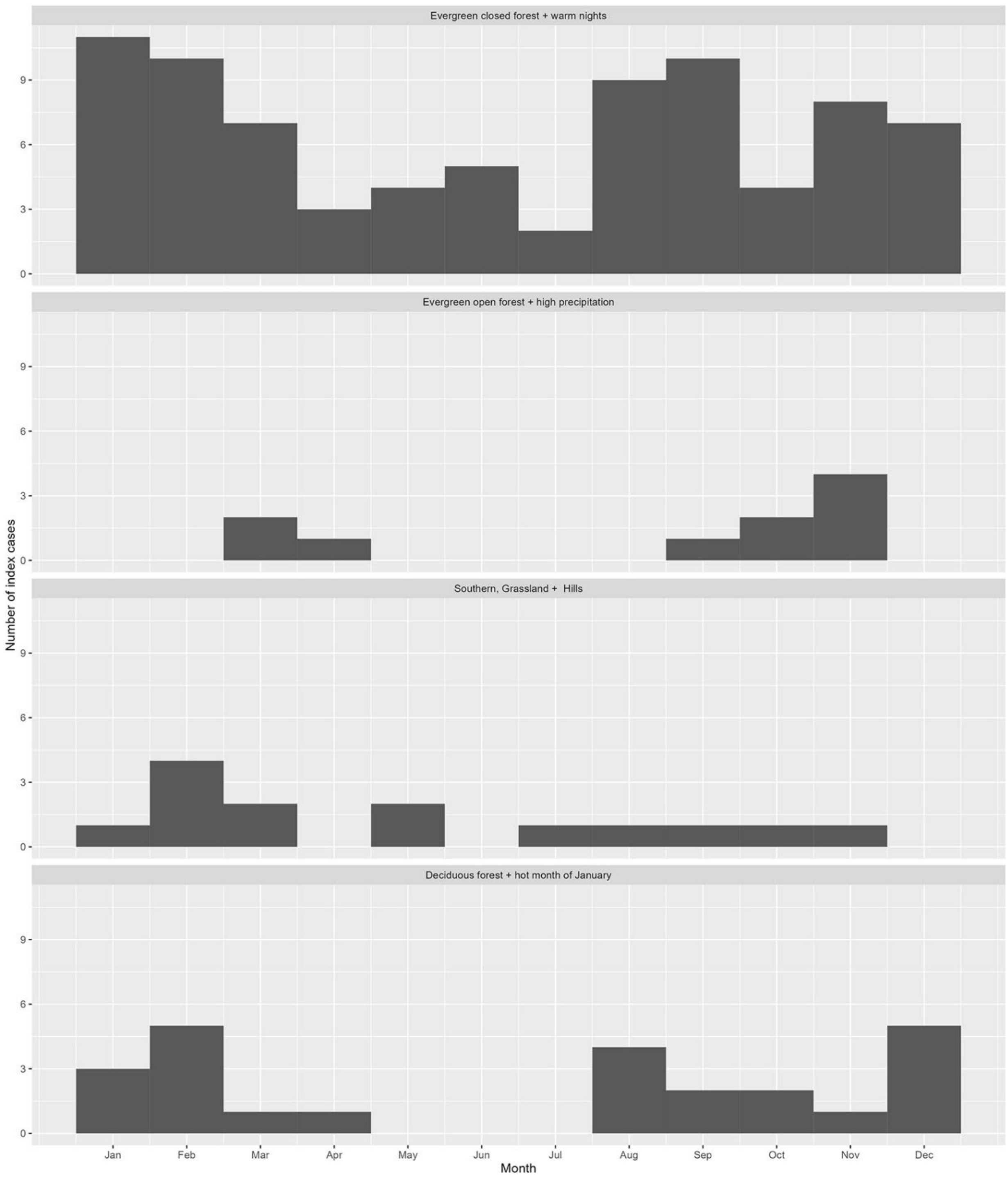

Appendix Figure 7. Distribution of mpox outbreaks by combined profile (landscape and months), n = 128 index cases (after excluding 4 high altitude and 1 Sahelian climate locations).

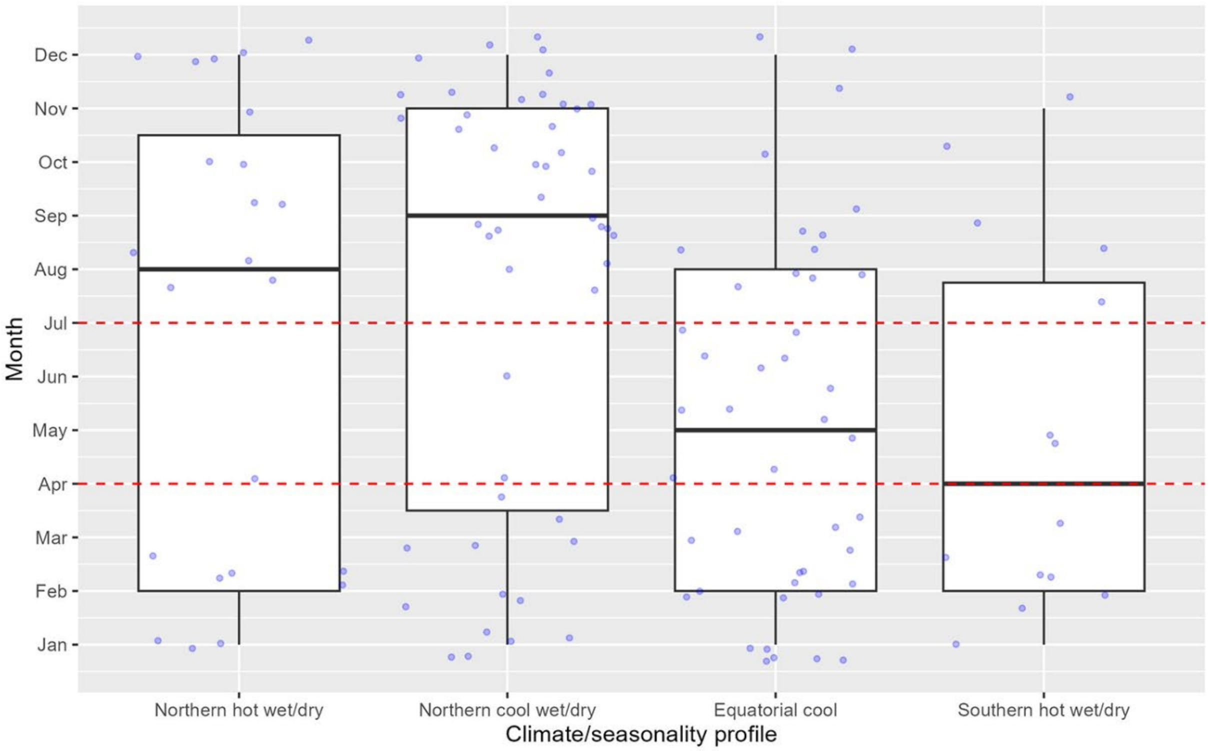

Appendix Figure 8. Boxplot presenting the distribution of months of index case according to the four climate/seasonality profiles. The red dashed lines indicate the low-risk period in the "Northern cool wet/dry" profile, while cases appear well distributed in the "Equatorial cool" profile. Kruskall-Wallis test p-value = 0.0042759 (Appendix Table 2).

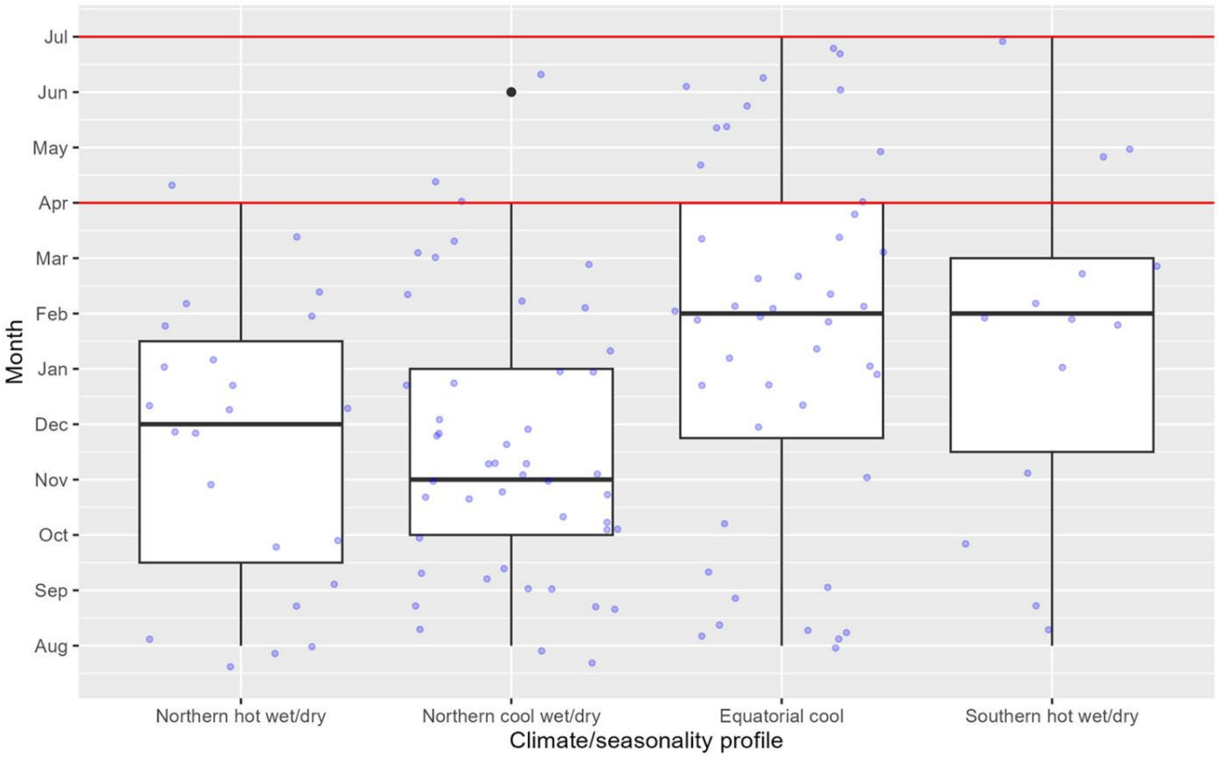

Appendix Figure 9. Boxplot presenting the distribution of months of index case according to the four climate/seasonality profile, starting from the start of the high-risk period and ending at the end of the low-risk period. Kruskall-Wallis test p value = 0.0047 (Appendix Table 3).

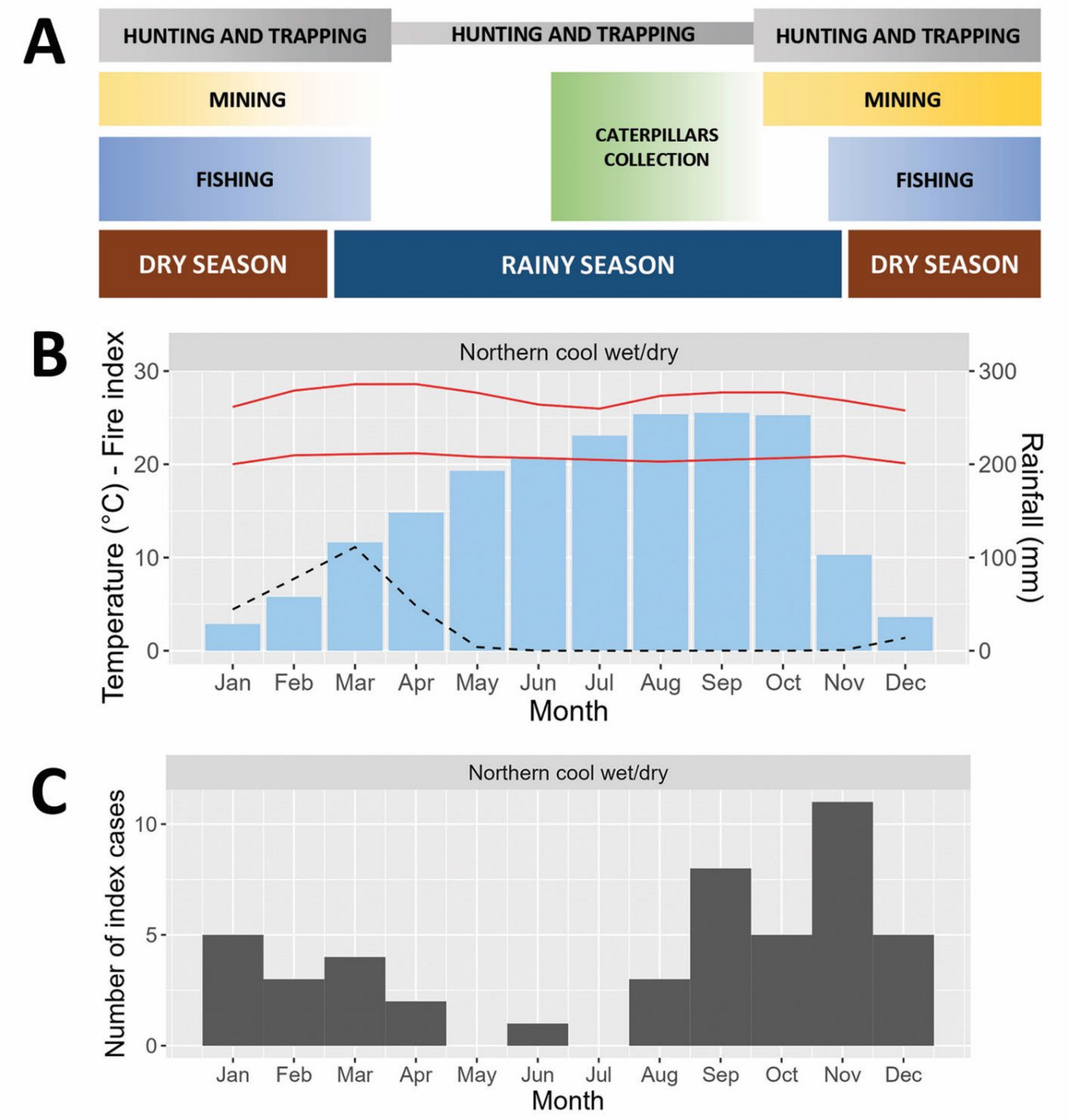

Appendix Figure 10. Schematic representation of possible links between seasonal activities, human exposures (A); climatic variations (B); and mpox zoonotic events (C) in the northern cool wet/dry climate.
